# Predicting Performance in Higher Education Using Proximal Predictors

**DOI:** 10.1371/journal.pone.0153663

**Published:** 2016-04-13

**Authors:** A. Susan M. Niessen, Rob R. Meijer, Jorge N. Tendeiro

**Affiliations:** Department of Psychometrics and Statistics, University of Groningen, Groningen, the Netherlands; University of Illinois-Chicago, UNITED STATES

## Abstract

We studied the validity of two methods for predicting academic performance and student-program fit that were proximal to important study criteria. Applicants to an undergraduate psychology program participated in a selection procedure containing a trial-studying test based on a work sample approach, and specific skills tests in English and math. Test scores were used to predict academic achievement and progress after the first year, achievement in specific course types, enrollment, and dropout after the first year. All tests showed positive significant correlations with the criteria. The trial-studying test was consistently the best predictor in the admission procedure. We found no significant differences between the predictive validity of the trial-studying test and prior educational performance, and substantial shared explained variance between the two predictors. Only applicants with lower trial-studying scores were significantly less likely to enroll in the program. In conclusion, the trial-studying test yielded predictive validities similar to that of prior educational performance and possibly enabled self-selection. In admissions aimed at student-program fit, or in admissions in which past educational performance is difficult to use, a trial-studying test is a good instrument to predict academic performance.

## Introduction

There is an increasing interest in the development of content-valid methods for prediction and selection in higher education [1]. Especially in many European countries where students apply to a specific study program rather than to a college, there is a trend towards selecting students based on admission tests that show correspondence to the program content. This trend is opposed to selecting students on the basis of more general admission criteria such as scores on general cognitive tests, personality questionnaires, or prior educational performance.

Tests that are used as predictors for study success and require similar skills for success as the criterion measures are proximal predictors. These proximal tests have been extensively studied in predicting job performance and were found to be among the most valid predictors [2]. Examples are job-knowledge tests, assessment centers, and work samples. In their meta-analysis Schmidt and Hunter [3] found that work sample tests were among the most valid test for predicting future job performance. However, despite the good results obtained in predicting job performance and the current use of such methods to select students for higher education in, for example, the Netherlands and Finland [4], they have hardly been studied empirically within the context of higher education.

The aim of this study was to fill this gap in the literature and to investigate the predictive validity of tests proximal to the criterion for predicting academic performance and student-program fit in an actual academic selection context. Most studies that investigate new methods to predict academic performance use data collected in low-stakes conditions (e.g. [1, 5]). We investigated the predictive validity of a trial-studying test, based on a work sample approach, and two specific skills tests for predicting academic performance in high-stakes selection procedure for a psychology program. Doing so, we provide empirical evidence that is badly needed to justify the use of these selection methods in institutes of higher education. The trial-studying test was designed to mimic a representative course in the program and the specific skills tests were designed to determine skills that were relevant for successful performance in specific courses.

### Proximal Predictors for Academic Performance

#### Specific skills tests

A limited amount of studies have been conducted in which the predictive validity of specific skills tests was investigated for predicting academic outcomes. Most studies were conducted in the context of predicting graduate school performance. Kuncel, Hezlett, and Ones [6] performed a meta-analysis across multiple disciplines and found that the specific subject tests of the Graduate Record Examinations were the best predictors for graduate school GPA in a study that also included verbal, quantitative and analytic ability, and undergraduate GPA. Furthermore, the specific subject tests alone predicted academic outcomes almost as well as composite scores of several general and subject-specific predictors. Kuncel et al. [6] explained these results through the similarity of the subject tests with the criteria used. Additionally, Kuncel and Hezlett [7] reviewed several studies and meta-analyses in predicting graduate school success and concluded that the strongest predictors were tests that were specifically linked to the discipline of interest.

#### Work sample tests

In behavioral prediction a distinction can be made between signs and samples as predictors of future behavior. Sign-based tests measure a theoretical construct (e.g., intelligence, personality) that is conceptually related to the criterion. Sample-based tests aim to sample behavior that is representative for the criterion behavior, based on the notion that current behavior is a good predictor for future behavior [8].

Tests for predicting educational performance have been mostly sign-based, measuring constructs such as cognitive abilities [9, 10]. However, Wernimont and Campbell [8] discussed that using behavioral sampling in prediction resulted in greater predictive validity than using signs of behavior. Also, Asher and Sciarrino [11] stated that the more a predictor and a criterion are alike, the higher the correlation is expected to be. “Information with the highest validity seems to have a point-to-point correspondence with the criterion” (p. 519).

Work sample tests are “high-fidelity assessment techniques that present conditions that are highly similar to essential challenges and situations on an actual job” [12] (p. 533) and meet the criteria of behavioral sampling and point-to-point correspondence. As discussed above, Schmidt and Hunter [3] also found in their meta-analysis that work sample tests were the best predictors of job performance. Callinan and Roberston [13] suggested that work samples perform well in predicting future performance because they measure a complex combination of individual abilities and skills that yield a higher validity than when these abilities and skills are measured separately. They also suggested that work samples contain a motivational component that is related to future performance. Some studies also suggested that work samples could enhance self-selection of applicants, both with respect to interests and abilities [14,15], and could therefore potentially reduce turnover. These characteristics make the work sample approach also appealing to use in admission to higher education. Trial-studying tests are based on the work sample approach applied in the context of higher education.

#### Trial-studying tests

Trial-studying tests are constructed as simulations of academic programs or representative parts of academic programs. We are aware of two studies that used trial-studying tests to predict performance in higher education [10,16]. Besides these two studies, there are a few studies about admission procedures for medical school that included similar methods [17,18], but they did not report validity coefficients so we do not discuss them here.

Lievens and Coetsier [10] studied a cohort of medical students and dental students who participated in an admission exam consisting of several cognitive tests, two trial-studying tests, and two situational judgment tests. They found that a cognitive reasoning test showed the largest relationship with first year mean grade, followed by the trial-studying tests, with medium-sized relationships. However, the reliabilities of the trial-studying tests were low, which likely had a negative influence on the estimated correlation coefficients. Visser, van der Maas, Engels-Freeke, and Vorst [16] studied a trial-studying test administered to select applicants for an undergraduate psychology program. The trial-studying test mimicked the first course in the program because results showed that the first grade obtained in higher education was a very good predictor for later academic performance [19]. Applicants who were rejected based on the test or had not participated in the selection procedure could still get admitted through a lottery procedure. Visser et al. [19] found that applicants admitted on the basis of the trial-studying test dropped out less often, earned higher grades, and obtained more course credit in the first year than applicants who were rejected by the test.

### Educational context

Proximal methods are particularly suitable when students apply directly to a program in a specific discipline, such as professional schools and graduate schools in the U.S. (like medical school or law school), and undergraduate programs and master programs in Europe. There are a number of reasons why especially the European higher education system is suitable for using proximal methods for selecting students. First, students often choose a specific program in which they major before starting undergraduate education, and they often apply directly to the educational program (e.g., medicine, psychology, or law). Second, many European countries have a certain degree of stratification in secondary education, with the best performing students attending the highest level of education. Only students that finished the appropriate secondary education program are eligible to apply to a university. In addition, graduation often depends on nationally or centrally organized final exams based on a national curriculum. Thus, there is a well-controlled central system and there is severe pre-selection on learning abilities for admission to higher education. This limits the utility of traditional predictors that measure general cognitive skills. Therefore, general cognitive tests are not often used. Finally, there is an increasing amount of international applicants (e.g. [20]), which makes it difficult to use previous educational performance as a selection criterion in practice. Graduation levels and diplomas are reasonably comparable across most countries, but high school grades are not because grading practices differ widely.

### Aims of the present study

The aim of the present study was to investigate the use of specific skills tests and the trial-studying test to predict performance in higher education and student-program fit. The trial-studying test was constructed to mimic the first courses in the program, so that the test had a high similarity to tasks that students are expected to perform. The specific skills tests were not designed to mimic the program, but covered specific subjects that were considered important for successful performance in specific courses. The tests were administered in a real selection procedure. We examined the predictive validity of these tests for first year academic performance and performance in types of specific course. In addition, we compared the predictive validity of these tests to that of prior educational achievement, one of the best general predictors for academic achievement in higher education (e.g. [21, 22]). Furthermore, we explored the presence of a self-selection effect as a result of the selection procedure and we investigated the relationship between test performance and dropout in the first year.

## Method

### Participants

The sample consisted of 851 applicants for an undergraduate psychology program in the academic year 2013–2014 at a Dutch university. All applicants participated in the selection procedure containing two specific skills tests and a trial-studying procedure. Of all applicants, 652 started the psychology program and 199 did not. The selection committee eventually rejected none of the applicants because the number of enrollments did not exceed the number of available places. Note that the students did not know this beforehand and thus the selection was high stakes and the applicants were likely to be very motivated to perform well. Sixty-nine percent of the applicants were female and the mean age was 20 for the entire applicant group (*SD* = 2.3) and also 20 (*SD* = 2.0) for the group that enrolled in the program. The students followed their courses in English or in Dutch, with similar content. The English program consisted of mainly international students. Fifty-six percent of the applicants followed the English program. Forty-three percent of all applicants were Dutch, 43 percent were German, 10 percent had another European nationality, and four percent had a non-European nationality.

### Materials and Procedure

#### Trial-studying test

The trial-studying test was designed to simulate a representative course in the first year. The psychology program requires a substantial amount of self-study and the students’ main tasks are studying books and syllabi, and attending lectures. However, attending the lectures is not mandatory. At the end of most courses, a multiple-choice exam is administered. To trigger future student-behavior, the trial-studying test mimicked the first course in the program, *Introduction to Psychology*. This course covered general psychological principles and theories. The students had to study a book and could attend lectures about the material before taking the exam. The applicants received two chapters from the book used in this course and were instructed to study them. One chapter was about research methodology, an important topic in this program, and one chapter was about more general psychological theories. The test consisted of 40 multiple-choice items and was constructed by a faculty-member who teaches first-year courses.

#### Skills tests

The applicants also completed specific skills tests in English reading comprehension and mathematics. English reading comprehension was included because most study material is in English, even in the Dutch program. The test consisted of 20 items and was constructed by a faculty member who is a professional translator. The items were fill-in-the gap exercises and questions about the meaning of the text. Mathematical skills were tested because the psychology curriculum includes a number of courses in statistics. The math skills included in the test were selected for their relevance to the statistics courses in the program. The test consisted of 30 items and was constructed by a faculty member who teaches first-year statistics courses. The applicants did not receive specific material to prepare for the specific skills tests, but useful webpages and example items were provided for the math test.

#### Selection procedure

After applying to the program, all applicants were invited to visit the university to take the admission tests. Each test had to be completed within 45 minutes with 15-minute breaks in between the tests. Proctors were present to prevent cheating. Applicants who had a valid reason for not being able to attend (living or working outside of Europe) could complete the admission tests online. Thirteen percent of the applicants used this option. Each test score was the sum of the number of items answered correctly. All applicants received feedback after a few weeks, including their scores on each test and a rank based on a composite score of the individual test scores. Students that held the lowest 165 ranks were contacted by phone and encouraged to rethink their enrollment, but this advice was not binding.

#### High school grades

In addition, high school grades were collected through the university administration for research purposes. High school grades were difficult to interpret across international students or students with an alternative educational background than the highest level of Dutch secondary education, due to different grading practices. Table 1 shows the sample sizes for each variable and for each combination of variables. The grades were self-reported but verified by the central education administration. Grades were on a scale of one to ten with ten being the highest score. We calculated a mean high school grade using the grades on all courses taken by a student, except courses that only provided a pass/fail result. The grade on a national final exam made up 50% of most final grades, the other 50% of the final grade was accounted for by exams administered by the schools in the last three years of secondary education. The inspectorate of education monitors the schools to prevent large differences in difficulty and quality. Thus, high school grades were fairly comparable across schools.

**Table 1:** Sample Sizes for Each Variable and Combinations of Variables in the Study, for Applicants Who Enrolled.

Variable		2.	3.	4.	5.	6.
1. Selection tests	652					
2. HSMG	203	203				
3. FC grade	626	198	215			
4. FYMG	638	201	625	638		
5. Credits	652	203	626	638	652	
6. Drop-out	652	203	626	638	652	652
7. SMG	590					
8. TMG	635					

Sample sizes for each variable are on the diagonal, sample sizes for combinations of variables are off-diagonal. HSMG, high school mean grade; FC grade, first course grade; FYMG, first year mean grade; SMG, statistics courses mean grade; TMG, theoretical courses mean grade.

#### Academic performance

Three measures of first-year academic performance were used: the first year mean grade for academic achievement, the number of obtained credits for academic progress, and the number of dropouts. Academic performance records were collected through the university administration after one academic year. Grades were on a scale of one to ten, with ten being the highest grade and a 6 or higher representing a pass. A first year mean grade (FYMG) was computed for each student, using the highest grade for each course after two exam opportunities (exam and resit) had taken place. One course only resulted in a pass/fail decision and was not taken into account. The FYMG consisted of 10 exam results when a student participated in all courses. Some students did start the program but did not participate in any exams. The resulting sample size for FYMG and combinations with other variables are shown in Table 1. Credit was granted after a course was passed and for most courses students earned five credit points, with a maximum of 60 credits in the first year, resulting in the first-year degree. Dropout students were also obtained from the administration.

Since the specific skills tests were designed to predict performance for certain types of courses, we also computed a composite mean grade for statistics courses (SMG) and theoretical courses (TMG). The SMG is the mean of the final grade for two statistics courses and the TMG is the mean final grade for seven courses that are concerned with psychological theory and require studying literature and completing an exam, without any practical components or assignments. Sample sizes for the number of students are also shown in Table 1.

Since we only used data available at the university, there were no manipulations in this study, and no identifiable information was presented, informed consent was not obtained. This was in line with the university’s privacy policy. This study was approved by and in accordance with the rules of the Ethical Committee Psychology from the University of Groningen [23].

### Procedure

Correlations were computed between the test scores and the academic performance measures. Effect sizes were interpreted according to Cohen’s [24] rules, although when interpreting effect sizes one should always take the context of the study into account. For significance tests we used α = .05. Before conducting the analyses, we conducted t-tests to check if there were test score differences between the applicants who took the tests online and those who took the tests proctored. We assumed that if the online applicants had cheated this would result in higher scores for these applicants as compared to those in the proctored group. For predictive validity we expected that scores on all tests would show significant positive relationships with all performance criteria, but that the trial-studying test would be the best predictor because it showed the most correspondence to the program.

To assess the validity of the trial-studying test, we assessed the relationships between the first course grade (*Introduction to Psychology*), the trial-studying test, and academic performance in the first year. For these analyses results from the first course were excluded from the FYMG and the number of obtained credits.

In addition, we assessed relationships between the test scores and achievement in specific course types, that is, the mean grade on the statistics courses, and the mean grade on the theoretical courses. For this purpose, multiple regression analyses were conducted with the test scores as independent variables and achievement in the courses as dependent variables. Squared semi-partial correlations were inspected to assess the unique contributions of the predictors. We expected that scores on the math test would be the strongest unique contributor to predicting the mean statistics grade, and that the trial-studying score would show the largest unique contribution to the mean theoretical grade, followed by the score on the English test.

To assess if the trial-studying test was a good alternative to using high school grades for applicants who completed Dutch secondary education, we compared the correlations of the trial-studying scores and academic performance with the correlations between HSMG and academic performance, using Williams test for differences between two dependent correlations [25]. We had no a priori expectation about the direction of these differences. In addition, we assessed the unique contributions of HSMG and the trial-studying score to predict academic performance. For FYMG and obtained credits as dependent variables, multiple regression analyses were conducted with the trial-studying score and high school grades as predictors. Squared semi-partial correlations were inspected to assess the unique contributions of both predictors. For dropout, a logistic regression analysis was conducted with, again, the trial studying scores and HSMG as predictors. As a proxy to semi-partial correlation in least-squared regression, pseudo-partial correlations, also known as Atkinson’s *R*, were computed and inspected [26]. While these coefficients cannot be directly compared to results obtained in least-squares regression, they do provide an indication of the contribution of each variable to the model.

Finally, we investigated whether the selection tests may have resulted in self-selection using logistic regression analyses with enrollment as the dependent variable and the test scores as independent variables, while controlling for receiving a phone call to encourage reconsidering enrollment. High school grades were not assessed for a self-selection effect, since they were not part of the admission procedure, the students received no feedback with respect to high school grades, and they were collected for research purposes only. Therefore, a self-selection effect based on high-school grades was unlikely to be expected.

## Results

### Predictive Validity

Before computing correlations between the test scores and academic performance, t-tests were conducted to check for differences in tests completed online or proctored. The applicants in the online test group obtained a lower mean score than the applicants in the proctored group for the trial-studying test and the English test and a higher mean score for the math test, but the latter difference was not significant (*t* (849) = 1.81, *p* = .07, Cohen’s *d* = 0.18). Based on these results there was no evidence that cheating seriously raised scores in the online group, and we merged the results for both groups together for all analyses. Descriptive statistics for the admission test scores, HSMG, academic performance, and the correlations between these variables are shown in Table 2. The reliability estimates of the admission tests were satisfactory and all admission tests showed significant correlations in the expected direction with all academic-performance criteria.

**Table 2:** Descriptive Statistics and Correlations Between the Predictors and Academic Performance Measures.

Variable	*M*	*SD*	1	2	3	4	5	6	7
*Admission tests*									
1. Trial-studying test	29.7	5.2	.81						
2. Math test	16.6	4.7	.30 _[.23,.37]_	.76					
3. English test	13.7	3.3	.43 _[.37,.49]_	.21 _[.14,.28]_	.70				
*Prior educational performance*									
4. HSMG	6.7	0.44	.45 _[.33,.55]_	.36 _[.23,.47]_	.29 _[16,.41]_				
*Academic performance*									
5.First course grade	6.6	1.4	.56 _[.50,.61]_	.20 _[.12,.27]_	.34 _[.27,.41]_	.55 _[.45,.64]_			
6. FYMG	6.6	1.3	.49 _[.43,.55]_	.29 _[.22,.36]_	.25 _[.18,.32]_	.52 _[.41,.61]_	.75^c^_[.71,.78]_		
7. Credits	46.0	20.2	.39 _[.32,.55]_	.20 _[.13,.27]_	.16 _[.09,.23]_	.30 _[.17,.42]_	.62^c^_[.57,.67]_	.82 _[.79,.84]_	
8. Drop-out^a^	0.20^b^		-.32 _[-.39,-.25]_	-.15 _[-.22,-.08]_	-.13 _[-.20,-.05]_	-.22 _[-.35,-.09]_	-.47 _[-.53,-.41]_	-.64 _[-.70,-.58]_	-.83 _[-.85,-.81]_

HSMG, high school mean grade; FYMG, first-year mean grade. Internal consistency coefficients are on the diagonal (Cronbach’s alfa). All correlations were significant with *p* < .01.

95% confidence intervals for the population correlation ρ are between brackets.

^a^Point-biserial correlations.

^b^Proportion.

^c^For these correlations, results on the first course were not included in the calculation of FYMG and credits.

The trial-studying test was the best predictor for all performance measures and showed a large effect size for FYMG (*r* = .49) and moderate effect sizes for obtained credits and dropout (*r* = .39 and *r* = -.32). The English test and the math test showed moderate effect sizes for achievement (*r* = .29 and *r* = .25) and small effect sizes for obtained credits and dropout (*r* ranging between -.13 and .20). Note that, as intended, the first course *Introduction to psychology* was strongly positively related to the trial-studying test (*r* = .56). Also, the grade in the first course was strongly related to all academic-performance criteria in the first year.

### Predictive validity for specific course achievement

Results of multiple regression analyses with the scores on the selection tests as independent variables and mean grades for statistics courses and theoretical courses as the dependent variables are shown in Table 3. The correlation between the mean grade on the statistics courses and the mean grade on the theoretical courses was *r* = .67, 95% CI [.62,.71], showing that they are strongly related but can be distinguished. Zero-order correlations between the selection test scores and specific course achievement were all positive and significant. For both specific course types, scores on the English test did not significantly contribute to the explained variance of the model when the trial-studying scores and the math scores were included. The trial-studying scores and the math scores predicted the mean statistics grade equally well with moderate effect sizes (*r* = .34, for both tests), and showed equal unique contributions to the model (*sr*^*2*^ = .07 for both tests). This only partly confirmed our expectations because we hypothesized that the math test would be the strongest predictor for statistics performance.

**Table 3:** Multiple Regression Results Predicting Specific Course Achievement with the Selection Test Scores.

Predictor	Course type					
	Statistics			Theoretical		
	β	*r*	*sr*^*2*^	β	*r*	*sr*^*2*^
Trial-studying score	.29*	.34*	.07*	.45*	.51*	.16*
Math score	.27*	.34*	.07*	.10*	.25*	.01*
English score	-.07	.11*	< .01	.06	.27*	< .01
*F*	44.22*			78.31*		
*R*^*2*^	.18			.27		

* *p* < .05

The trial-studying score showed a large positive relationship with the mean theoretical grade (*r* = .51) and the math score and the English score showed small to moderate positive relationships (*r* = .25 and *r* = .27). The unique contribution was the largest for the trial-studying scores (*sr*^*2*^ = .16) and very small to non-existent for the math scores and the English scores. This also partly confirmed our expectations, since a unique contribution of the English scores was expected.

### Comparing trial-studying to prior educational achievement

For applicants who completed Dutch secondary education, the mean high school grade also showed significant correlations with all academic-performance criteria, with a large effect size for FYMG (*r* = .52), a moderate effect size for obtained credits (*r* = .30), and a small effect size for dropout (*r* = -.22). To compare the predictive validities of HSMG and the trial-studying test we computed the correlations again for only the students with available data for HSMG, the trial-studying test, and FYMG (*n* = 201), for HSMG, the trial-studying test and obtained credits (*n* = 203), and for HSMG, the trial-studying test and dropout (*n* = 203). The correlations between the trial-studying score and the academic performance measures were slightly lower within this group than for the entire sample (FYMG, *r* = .41, credits, *r* = .29, and dropout, *r* = -.26). Taking into account the correlation between the trial-studying score and HSMG (*r* = .45), the results of William’s test showed no significant difference between the predictive validity of the trial-studying score and HSMG for FYMG, with *t*_*(198)*_ = -1.75, *p* = .08. There was also no significant difference in predictive validity for obtained credits, with *t*_*(200)*_ = -0.14, *p* = .89, and no significant difference in predictive validity for drop-out, with *t*_*(200*)_ = -0.56, *p* = .58.

To assess the unique contributions and overlap between these two predictors for academic achievement (FYMG) and progress (obtained credits) in the first year, multiple regression analyses were conducted and semi-partial correlations were assessed. For FYMG the model was significant (*F*_*(2*,*198)*_ = 44.45, *p* < .01 and *R*^*2*^ = .31), and the unique contribution for HSMG was *sr*^*2*^ = .15 and for the trial-studying test it was *sr*^*2*^ = .04. Hence, the shared explained variance for FYMG by HSMG and the trial-studying score equaled .12. Thus, for applicants with Dutch secondary education, HSMG uniquely explained more variance in the FYMG than the trial-studying score, whereas they also shared a substantial part of explained variance. For obtained credits the model was also significant (*F*_*(2*,*200)*_ = 14.02, *p* < .01 and *R*^*2*^ = .12), with *sr*^*2*^ = .04 for HSMG and *sr*^*2*^ = .03 for the trial-studying score. The shared explained variance for obtained credits by HSMG and the trial-studying score equaled .05. The uniquely explained variance for each predictor and the shared explained variance for obtained credits were of similar magnitude.

For dropout as the dependent variable, the logistic regression model with HSMG and the trial-studying score as independent variables was significant (χ^2^(2) = 17.02, *p* < .01, and Nagelkerke’s pseudo *R*^*2*^ = .13). Pseudo-partial correlations equaled *pr* = .09 for HSMG and *pr* = .13 for the trial-studying score. Thus, the unique contribution of the trial-studying test when taking HSMG into account was slightly larger than vice versa.

### Self-selection

Descriptive statistics for enrolled and not-enrolled applicants are presented in Table 4. Enrollment was predicted based on the selection test scores using logistic regression analysis, controlling for receiving a phone call to reconsider enrollment after scoring among the 165 lowest ranks. Results for the logistic regression analysis are in Table 5. The model was significant for predicting enrollment. The trial-studying score was the only significant predictor in the model, showing a small effect. A one-unit increase in the trial-studying score increases the odds of enrolling by a factor of 1.02 to 1.09, when the other test scores and receiving a discouraging phone call were ‘held constant’.

**Table 4:** Means and Standard Deviations for Applicants Who Did Enroll and Did Not Enroll in the Program.

Variable	Enrolled	Not enrolled
Phone call^a^	.14	.35
Trial-studying test	29.7 (5.2)	27.0 (6.5)
Math test	16.6 (4.7)	14.9 (4.7)
English test	13.7 (3.3)	12.6 (3.6)

Standard deviations are between brackets.

^a^ Proportion of students who received a discouraging phone call within the enrolled and non-enrolled group.

**Table 5:** Logistic Regression Results for Predicting Enrollment Based on Selection-test Scores.

Variables		*B*	*SE (B)*	Wald *X*^*2*^	*df*	*p*	*e*^*B*^	95% CI *e*^*B*^
Phone call		.53	.29	3.38	1	.07	1.70	0.97,2.99
Trial-studying score		.05	.02	7.67	1	.01	1.05	1.02,1.09
Math score		.03	.02	1.67	1	.20	1.03	0.77,1.07
English score		.01	.03	0.06	1	.80	1.01	0.95,1.07
Model *X*^*2*^	46.44				4	< .01		
*n*	851							

## Discussion

The results of this study showed that all proximal tests predicted academic performance in the first year. The predictive validity of the trial-studying test was moderate to large for academic performance in the first year, whereas the predictive validities for the specific skills tests were small to moderate. The results also showed that the first course in the program was a very good predictor for performance in the rest of the first year, replicating results by Colenbrander and Vorst [19]. Furthermore, scores on the trial-studying test were related to student-program fit, as shown by a moderate relationship with dropout and a small but significant relationship with enrollment decisions.

The specific skills tests did not predict achievement in specific related course types better than the other tests. An interesting result was that the trial-studying test predicted achievement in statistics courses equally well as the math test and that the English test was not a better predictor than the math test for the grades in theoretical courses. A possible explanation for these results is that the trial-studying test, following a work sample approach, measures both ability and motivation to perform well [13]. This implicit behavioral measurement of motivation could explain the relationships between the trial-studying test and academic performance, even when the course content was different from the trial-studying test. After all, motivation and effort are necessary for successful performance in any course. As de Raad and Schouwenburg [27] stated ‘achievement through ability alone is the exception rather than the rule’. Lievens and Coetsier [10] found lower predictive validities using a similar work sample approach than we did, but their work samples had relatively low reliability and they were not specifically designed to mimic relevant parts of the programs. Compared to prior educational performance as measured by mean high school grades, which is one of the most established predictors of academic performance in higher education, the trial-studying test showed lower predictive validity for applicants who completed Dutch secondary education. However, the differences were not significant. Additionally, the regression results obtained using this subsample showed that the trial-studying score and HSMG shared a substantial proportion of explained variance in academic performance. The HSMG uniquely explained more variance in achievement, and the trial-studying test had a slightly larger unique contribution to predicting dropout.

It is important to note here that although HSMG is a good predictor for academic performance, the drawback in practice is that these grades are not always available and/or are difficult to compare across candidates, as we explained above. Furthermore, an advantage of using proximal tests is the high content validity. These tests could help provide insight in what the study program is like, and what is expected of the applicants when they are accepted as students. This could result in a self-selection effect, and our results showed that applicants with lower scores on the trial-studying test were significantly less likely to enroll in the program, even after controlling for actively discouraging low-scoring applicants to enroll. However, the effect was small and we do not know if the decision to enroll or not was based on the experience or results of the admission procedure. It is possible that applicants who decided not to enroll were already less motivated or uncertain about their choice, and did not prepare well for the tests as a result. Another advantage of proximal tests is that candidates are not ‘haunted by their past’. In contrast to HSMG, which are fixed and cannot be altered by the candidates, proximal tests provide candidates an opportunity to show their ability and motivation for the study program.

### Limitations

In this study we used a sample of applicants from one cohort of students in one discipline, and obtained criterion measures after one academic year. Although strong relationships were found in previous studies between academic performance in the first year and in later years [28] data that provide insight in the predictor-criterion relations for a longer period of time should be collected. The predictive validity is expected to decrease somewhat when academic performance is measured with a larger time interval.

Prior educational performance could only be studied for candidates who applied to the program after completing Dutch secondary education at the level that traditionally allows admission to universities. Approximately two-thirds of the students had a different educational background. Data on prior educational performance are difficult to compare for these applicants. However, this also illustrates that using prior educational performance, as an admission tool, is difficult to realize in practice.

Furthermore, constructing a proximal test for programs like psychology is relatively straightforward, but for some programs it may be more challenging. Many academic undergraduate programs, like psychology, require mostly independent studying, attending lectures, and completing exams. However, there are programs that are more directed towards the mastery of practical skills. For example in medical school, teacher training, or vocational education, skills such as motor skills or communication skills may have predictive value. These skills are more complicated to assess. In addition to a trial-studying test measuring ‘classic’ student behavior like studying literature, proximal methods can also be used to measure non-academic skills. An example is the multiple mini-interview (MMI) used to assess applicants to medical school. The MMI can be used to assess applicants on moral reasoning, communication skills, and social skills. MMI scores predicted clerkship performance [29,30] and performance on clinical skills examinations [30,31]. Lievens [32] found that scores on SJT’s used to select applicants for medical school predicted especially the more practical and interpersonally oriented outcomes, whereas cognitive (skills) tests predicted those outcomes to a lesser or no extent. However, effect sizes were mostly small to moderate and based on data obtained in low-stakes conditions [33].

Also, if trial-studying tests are constructed in accordance with the test in this study, a unique test has to be constructed for each program, preferably with new items each time the test is administered. Standardized tests are usually carefully constructed, analyzed, and checked with respect to difficulty level and psychometric quality. Constructing this trial-studying test was not more time-consuming than constructing a typical exam. However, a potential drawback is the risk of unsatisfactory test-quality. Close attention should be paid to characteristics such as difficulty, item quality, and reliability.

Finally, our results showed that the predictors in this study explained 10% through 25% of the variance depending on the outcome measure and predictor. This may seem low to some critics. However, it is good to remember that, as for example, Dawes [34] argued, many critics implicitly assume that the remaining 90% through 75% of the variance can be explained. Considering the complex nature of the outcomes that we want to predict (that is, student performance in the future), we may not expect much better results. Indeed, in the context of predicting academic performance, the highest predictive validities found in many studies are around *r* = .50 after correcting for range restriction and unreliability [6, 7]. The value of using admission tests is that they do improve selection decisions as compared to not using these tests.

### Conclusion

This study showed that a work sample approach can be implemented successfully in the context of higher education. Also, proximal methods tend to have high content validity due to the similarity to the criterion [2]. A question that can be addressed in the future is whether the favorable characteristics of proximal approaches and in particular work sample approaches found in research in personnel selection, such as perceived fairness and face validity [35] also extend to an educational context.

In our study, both prior educational performance and the trial-studying test yielded moderate to large predictive validity, whereas the specific skills test showed smaller effects. When information about prior educational performance is available, comparable, and verifiable for the majority of applicants, this information may be the most effective and efficient approach to select candidates. When this is not the case, using a trial-studying test is a good alternative and may be preferred over specific skills tests. Contexts in which proximal tests could be preferred over traditional admission criteria are admission procedures with an emphasis on assessing student-program fit that aim for high content validity. An example is the mandatory matching procedure in the Netherlands. Applicants to open-admission programs are required to participate in a ‘matching’ procedure organized by the individual study programs. The result is a non-binding advice about enrollment based on student-program fit. When constructing trial-studying tests for other programs, we recommend to start with an analysis of the study program and to identify representative courses for the program that show a high relationship with performance in the rest of the program.

## Supporting Information

S1 Data Dataset Psychology Applicants. Uncommon values were merged to categories to assure anonymity of the participants.
(SAV)
